# Endosymbiotic bacteria of the boar louse *Haematopinus apri* (Insecta: Phthiraptera: Anoplura)

**DOI:** 10.3389/fmicb.2022.962252

**Published:** 2022-08-08

**Authors:** Yudai Nishide, Kohei Oguchi, Maria Murakami, Minoru Moriyama, Ryuichi Koga, Takema Fukatsu

**Affiliations:** ^1^Institute of Agrobiological Sciences, National Agriculture and Food Research Organization (NARO), Tsukuba, Japan; ^2^National Institute of Advanced Industrial Science and Technology (AIST), Tsukuba, Japan; ^3^Misaki Marine Biological Station, School of Science, The University of Tokyo, Miura, Japan; ^4^Department of Biological Sciences, Graduate School of Science, The University of Tokyo, Tokyo, Japan; ^5^Graduate School of Life and Environmental Sciences, University of Tsukuba, Tsukuba, Japan

**Keywords:** *Haematopinus apri*, boar louse, *Haematopinus suis*, hog louse, symbiont, evolution, domestication, symbiotic organ

## Abstract

Insects exclusively feeding on vertebrate blood are usually dependent on symbiotic bacteria for provisioning of B vitamins. Among them, sucking lice are prominent in that their symbiotic bacteria as well as their symbiotic organs exhibit striking diversity. Here we investigated the bacterial diversity associated with the boar louse *Haematopinus apri* in comparison with the hog louse *Haematopinus suis*. Amplicon sequencing analysis identified the primary endosymbiont predominantly detected from all populations of *H. apri* with some minor secondary bacterial associates. Sequencing and phylogenetic analysis of bacterial 16S rRNA gene confirmed that the endosymbionts of the boar louse *H. apri*, the hog louse *H. suis* and the cattle louse *Haematopinus eurysternus* form a distinct clade in the Gammaproteobacteria. The endosymbiont clade of *Haematopinus* spp. was phylogenetically distinct from the primary endosymbionts of other louse lineages. Fluorescence *in situ* hybridization visualized the endosymbiont localization within midgut epithelium, ovarial ampulla and posterior oocyte of *H. apri*, which were substantially the same as the endosymbiont localization previously described in *H. suis* and *H. eurysternus*. Mitochondrial haplotype analysis revealed that, although the domestic pig was derived from the wild boar over the past 8,000 years of human history, the populations of *H. apri* constituted a distinct sister clade to the populations of *H. suis*. Based on these results, we discussed possible evolutionary trajectories of the boar louse, the hog louse and their endosymbionts in the context of swine domestication. We proposed ‘*Candidatus* Haematopinicola symbiotica’ for the distinct clade of the endosymbionts of *Haematopinus* spp.

## Introduction

Insects represent the majority of the biodiversity in the terrestrial ecosystem (Grimaldi and Engel, 2005), in which symbiotic microorganisms generally play important biological roles (Buchner, 1965; Bourtzis and Miller, 2003). In particular, insects utilizing nutritionally recalcitrant food resources are usually dependent on their specific microbial partners, wherein the representative microbial functions are digestion of plant cell wall polymers that are otherwise indigestible for many wood-feeding insects like termites, bark beetles, etc. (Brune, 2014; Biedermann and Vega, 2020), provisioning of essential amino acids that are lacking in plant vascular fluid for plant sap-sucking insects like aphids, cicadas, etc. (Moran et al., 2008; Douglas, 2009), and the synthesis of B vitamins that are deficient in vertebrate blood for blood-sucking insects like lice, bedbugs, etc. (Rio et al., 2016; Husnik, 2018).

Blood-sucking insects, such as mosquitoes, fleas, tsetse flies, lice, bedbugs, etc., are notorious not only for causing itch, wound, and inflammation to human skin but also for vectoring devastating human and veterinary pathogens (Lehane, 2005). Among them, those feeding on vertebrate blood throughout their life stages usually develop specialized symbiotic organs for hosting specific symbiotic bacteria: *Wigglesworthia* in tsetse flies (Aksoy, 1995; Akman et al., 2002), *Aschnera* in nycteribiid bat flies (Hosokawa et al., 2012), *Wolbachi*a in bedbugs (Hosokawa et al., 2010; Nikoh et al., 2014), and others.

Sucking lice (Phthiraptera: Anoplura) live on vertebrate blood as the sole food source throughout their life cycle (Lehane, 2005), and most of them develop specialized symbiotic organs that harbor specific symbiotic bacteria (Ries, 1931; Buchner, 1965). Both histological inspection (Ries, 1931) and molecular phylogenetic survey (Hypša and Křížek, 2007) revealed that their symbiotic organs and associated bacterial symbionts are strikingly diverse among different lice lineages and likely of independent evolutionary origins: ‘*Candidatus* Riesia spp.’ are harbored in a distinct oval symbiotic organ located on the ventral side of the intestine called the “stomach disc” in human and primate lice *Pediculus* spp. and *Pthirus* spp. (Sasaki-Fukatsu et al., 2006; Allen et al., 2007; Perotti et al., 2007; Kirkness et al., 2010; Boyd et al., 2014); ‘*Candidatus* Puchtella pedicinophila’ is harbored in a specific region of the midgut epithelium in the monkey lice *Pedicinus* spp. (Fukatsu et al., 2009; Boyd et al., 2017); and ‘*Candidatus* Legionella polyplacis’ is in an intestine-associated symbiotic organ in a rodent louse *Polyplax serrata* (Říhová et al., 2017). In other lice lineages, the symbiotic organs are histologically not so distinct and the symbiotic associations might be either younger and/or casual: *Sodalis* and *Rickettsia* in a seal louse *Proechinophthirus fluctus* (Boyd et al., 2016); *Neisseria/Snodgrassella*-allied in rodent lice *Hoplopleura* spp. and *Polyplax* spp. (Říhová et al., 2021). Thus far, however, *in vivo* localization and developmental dynamics of the symbiotic organs and the symbiotic bacteria have been only briefly described in most cases, except for those in the human body louse *Pediculus humanus* and also those in the cattle louse *Haematopinus eurysternus* (Ries, 1931; Buchner, 1965).

The genus *Haematopinus* consists of some 20 species of ungulate lice, including the cattle louse *H. eurysternus*, the hog louse *Haematopinus suis*, the horse louse *Haematopinus asini*, and others (Durden and Musser, 1994). Historically, the endosymbiotic bacteria of *Haematopinus* spp. have been described as follows: the initial microscopic detection in *H. suis* (Sikora, 1919; Buchner, 1920; Florence, 1924); detailed histological inspection of *in vivo* localization and developmental dynamics in *H. eurysternus* (Ries, 1931); transmission electron microscopic observation in *H. suis* (Żelazowska and Biliński, 1999); and molecular phylogenetic analysis based on 16S rRNA gene sequences from *H. suis*, *H. eurysternus* and *Haematopinus apri* (Hypša and Křížek, 2007). These previous studies, mostly conducted on *H. suis* or *H. eurysternus*, have shown that the endosymbionts of *Haematopinus* spp. form a well-supported clade in the Gammaproteobacteria and exhibit peculiar localization patterns distinct from the other louse lineages: in both females and males, numerous bacteriocytes are scattered over the midgut epithelium, recognized as swellings into the midgut cavity, in which the symbiotic bacteria look like endocellular at a glance but actually reside in an extracellular space surrounded by the bacteriocyte cytoplasm; and specifically in females, the symbiotic bacteria also localize to the dorsal “depot bacteriomes,” from which the symbiotic bacteria migrate to the ovarial ampullae and infect to developing oocytes (Ries, 1931; Buchner, 1965). These early histological works, based on conventional light microscopy and illustrated by hand-drawn sketches, should be re-examined by modern histological techniques and sophisticated microscopy.

The boar louse *H. apri* parasitizes the wild boar and distributes across Eurasia (Durden and Musser, 1994). Reflecting the recent origin of the pig (*Sus scrofa domestica*) *via* domestication of the wild boar (*Sus scrofa scrofa*) over the past 8,000 years (Larson et al., 2005; Larson and Fuller, 2014; Frantz et al., 2016), the boar louse *H. apri* is morphologically and phylogenetically similar to the hog louse *H. suis*, although the former exhibits paler body color, relatively smaller legs, and smaller paratergites in comparison with the latter (Ferris, 1933). As for the endosymbiont of *H. apri*, only a bacterial 16S rRNA gene sequence (1,213 bp in size; DQ076665), has been reported and analyzed for inferring the molecular phylogenetic placement (Hypša and Křížek, 2007), while no other biological information is available. The evolutionary relationship between *H. apri* and *H. suis* is somewhat reminiscent of the relationship between the human head louse (*P. humanus capitis*) and the human body louse (*P. humanus humanus*) that has been argued in relation to human clothing and civilization (Kittler et al., 2003; Veracx and Raoult, 2012; Amanzougaghene et al., 2020). In this context, detailed characterization of the endosymbiont of *H. apri* in comparison with the endosymbiont of *H. suis* is of interest.

In an initial attempt toward this research goal, we investigated the bacterial diversity associated with multiple populations of the boar louse *H. apri* using amplicon sequencing, molecular phylogenetic and histological approaches, thereby identifying the primary endosymbiont and minor bacterial associates of *H. apri*. We discussed the relationship between the endosymbionts of *H. apri* and *H. suis* in the context of swine domestication, and proposed a candidate name for the endosymbiotic bacteria associated with the louse genus *Haematopinus*.

## Materials and methods

### Insect materials

Table 1 and Supplementary Table S1 list the insect samples examined in this study. These insects were preserved either in ethanol or acetone (Fukatsu, 1999), or brought to the laboratory alive and processed immediately. For histological observations, the insects were dissected in phosphate-buffered saline (PBS; 0.8% NaCl, 0.02% KCl, 0.115% Na_2_HPO_4_, 0.02% KH_2_PO_4_) by using fine tweezers under a dissection microscope (M165FC; Leica).

**Table 1:** Samples of the boar louse *H. apri* and the hog louse *H. suis* examined in this study.

Louse species	Host animal	Collection locality^1^	Collection date	Collector^2^	Sample ID^3^	Note
Boar louse	Wild boar					
*Haematopinus apri*	*Sus scrofa leucomystax*	Toyama	Feb. 2008	TY	#01-#18	Fixed specimens
		Wakayama	Feb.-Mar. 2008	AT	#19-#33	Fixed specimens
		Oita	Mar. 2008	AT	#34-#41	Fixed specimens
		Hyogo	Nov. 2008, Mar. 2009	AT	#42-#50	Fixed specimens
		Osaka	Mar. 2008	SU	#51-#56	Fixed specimens
		Ehime	Dec. 2021	YM	#57-#58 + α	Fresh specimens
Hog louse	Domestic pig					
*Haematopinus suis*	*Sus scrofa domestica*	Saitama	Jun. 2008	K	#59-#63	Fixed specimens

^1^All localities are in Japan.

^2^AT, Ai Takano; K, Kato; SU, Shigehiko Uni; TY, Takeo Yamauchi; YM, Yuki Miyaoka.

^3^Detailed sample information and associated sequence accession numbers are described in Supplementary Table S1.

### DNA extraction, PCR, cloning and sequencing

The samples were washed by soaking in 1% bleach and 0.1% Triton X-100 for 1 min and then rinsed twice with water. Then, the samples were individually crushed using BioMasher II (Nippi, Tokyo, Japan) and subjected to DNA extraction using DNeasy Blood and Tissue kit (Qiagen, Hilden, Germany). Insect mitochondrial cytochrome c oxidase subunit I (COI) gene was amplified by PCR using KOD FX Neo (TOYOBO, Osaka, Japan) with the primers modified-mtd6 (5′-GGA GGW TTY GGA AAT TGR TTA GTD CC-3′) and mtd11 (5′-ACT GTA AAY ATA YGR TGW GCT CA-3′) (Jiang et al., 2013) that were selected to match COI sequences of *H. suis* and *H. apri* (accession numbers HM241908 and KC814616). Bacterial 16S rRNA gene was amplified by PCR using Ex taq HS (TaKaRa, Shiga, Japan) with two sets of the primers 16SA1 (5′-AGA GTT TGA TCM TGG CTC AG-3′)—16SB2 (3′-CGA GCT GAC ARC CAT GCA-3′) and 16SA2 (5′-GTG CCA GCA GCC GCG GTA ATA C-3′)—16SB1 (5’-TAC GGY TAC CTT GTT ACG ACT T-3′) (Fukatsu and Nikoh, 1998). The PCR products were cloned using pGEM-T Easy Vector (Promega, Madison, United States), Mighty mix for DNA ligation (Takara) and *Escherichia coli* DH5 competent cells (Takara). The inserted plasmids were extracted from the transformed *E. coli* cells using QIAprep Spin miniprep kit (QIAGEN) and subjected to sequencing reactions using BigDye Terminator v3.1 Cycle Sequencing Kit (Thermo Fisher Scientific, Massachusetts, United States) with the primers M13FW (5′-GTA AAA CGA CGG CCA GT-3′) and M13RV (5′-CAG GAA ACA GCT ATG AC-3′) targeting the flanking regions of the vector.

### Molecular phylogenetic analysis

The molecular phylogenetic analyses were conducted by the maximum likelihood methods using MEGA 5.2 (Tamura et al., 2011). The optimum model was selected by model tests to describe each phylogenetic tree. Bootstrap probability values based on 1,000 replications are shown on the nodes. To analyze the genetic diversity of host COI sequences, we depicted the network diagrams of COI haplotypes using MEGA-X (Kumar et al., 2018), DnaSP 6 (Rozas et al., 2017) and Network 10 (fluxus-engineering.com).

### Amplicon sequencing

The hypervariable V3/V4 region of bacterial 16S rRNA gene, around 0.4 kb in size, was targeted for amplicon sequencing analyses. Library construction was conducted by the 2-step tailed PCR procedure with the primers first_341_MIX (5′-ACA CTC TTT CCC TAC ACG CTC TTC CGA TCT NNN NNC CTA CGG GNG GCW GCA G-3′) and first_805r_MIX (5′-GTG ACT GGA GTT CAG ACG TGT GCT CTT CCG ATC TNN NNN GAC TAC HVG GGT ATC TAA TCC-3′), and then second_F (5′-AAT GAT ACG GCG ACC ACC GAG ATC TAC AC-Indexseq (8mer)-ACA CTC TTT CCC TAC ACG ACG C-3′) and second_R (5′-CAA GCA GAA GAC GGC ATA CGA GAT-Indexseq (8mer)-GTG ACT GGA GTT CAG ACG TGT G-3′). The libraries were measured for concentration using Synergy H1 (Bio Tec, Vermont, United States) and QuantiFluor dsDNA System (Promega), and quantified using Fragment Analyzer (Agilent, California, United States) and dsDNA 915 Reagent Kit (Agilent). Sequencing of the libraries was performed using Miseq (Illumina, California, United States). From the raw amplicon sequences obtained (accession number DRR360832-DRR360847), only the reads containing the tags in PCR primers were selected using FASTX-Toolkit (ver.0.0.14). The output reads were subjected to denoising and removal of chimeric sequences using the DADA2 plugin in the Quantitative Insights Into Microbial Ecology (QIIME2) v.2021.11 pipeline (Bolyen et al., 2019). Bacterial taxa were assigned to the representative amplicon sequences based on GreenGenes V.13_8 (McDonald et al., 2012) using the q2-feature-classifier QIIME2 plugin (Bokulich et al., 2018).

### Fluorescence *in situ* hybridization (FISH)

Whole-mount fluorescent *in situ* hybridization (FISH) targeting bacterial 16S rRNA was performed essentially as described previously (Koga et al., 2009). The dissected insect tissues were fixed in 4% paraformaldehyde in PBS for 3 h and then thoroughly washed in PBST (0.1% Tween 20 in PBS). The fixed insect tissues were washed twice in hybridization buffer (20 mM Tris–HCl [pH 8.0], 0.9 M NaCl, 0.01% SDS, 30% formamide). To specifically target 16S rRNA of the primary symbiont of *Haematopinus* spp., we designed the oligonucleotide probe Wbsym1181R (5′-ACC TTC GCA GGT TAG CTT-3′) labeled with fluorochrome Alexa Fluor 647 at the 5′ terminus. For universal detection of bacterial 16S rRNA, we used the probe EUB338 (5′-GCT GCC TCC CGT AGG AGT-3′) (Amann et al., 1990) labeled with Alexa Fluor 555 at the 5′ terminus. The samples were incubated in hybridization buffer containing 50 nM probe. After washed twice in PBST, host nuclear DNA and filamentous actin were stained with 4.5 μM 4′,6-diamidino-2-phenylindole (DAPI; Thermo Fisher Scientific) and 1.5 μM Alexa Fluor 488-labeled phalloidin (Thermo Fisher Scientific), respectively, for 1 h at room temperature. Then, the samples were washed with PBST again, mounted in 50% glycerol in PBS, and observed under a laser scanning confocal microscope (LSM 700; Carl Zeiss, Germany). As a control experiment for FISH detection, RNase digestion controls in which the tissue samples were treated with RNase A prior to hybridization were conducted (Supplementary Figure S1).

## Results

### Microbiome of *Haematopinus apri* and *Haematopinus suis*

**Figure 1:**
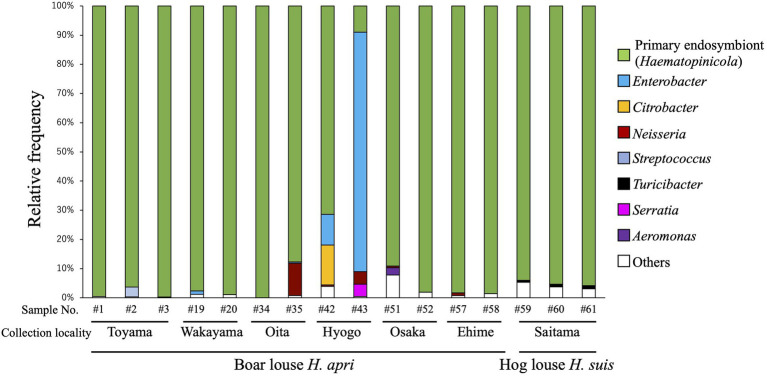
Bacterial diversity associated with the boar louse *Haematopinus apri* and the hog louse *Haematopinus suis* based on amplicon sequencing data of the hypervariable V3/V4 region of 16S rRNA gene. Assigned bacterial taxa are color-coded as shown on the right side. As for sample information and amplicon sequencing data, see Supplementary Table S1.

In order to grasp the bacterial diversity associated with *H. apri*, 13 insects representing six collection localities (see Supplementary Table S1) were subjected to amplicon sequencing analysis targeting the hypervariable V3/V4 region of bacterial 16S rRNA gene. In addition, three insects of *H. suis* collected from a locality were analyzed in the same way (see Supplementary Table S1). Figure 1 shows the composition of the amplicon sequences for each of the insect samples. The same bacterial taxon was predominantly detected from almost all the samples (except for Hyogo samples of *H. apri*), accounting for over 85% of the detected sequences for each of the samples. The remaining minor bacterial taxa were assigned as allied to *Neisseria*, *Streptococcus*, *Serratia* and *Aeromonas* for *H. apri*, and *Turicibacter* for *H. suis* (Figure 1). Exceptionally, two Hyogo samples of *H. apri* exhibited different patterns: in a sample, *Enterobacter* was highly predominant, accounting for over 80% of the detected sequences; in another sample, not only *Enterobacter* but also *Citrobacter* occupied substantial fractions of the microbiota (Figure 1). Notably, these Hyogo samples were shipped to our laboratory by refrigerated, not frozen, courier service, delayed in arrival, and then transferred to an ethanol vial for preservation. Hence, it seems likely that proliferation of the microbes in the dead insect bodies resulted in the exceptional microbial compositions in these samples. Taken together, these results strongly suggested that a single bacterial species constitutes the major microbial associate of *H. apri* and *H. suis*, which is likely the primary endosymbiont as described in previous histological and phylogenetic studies on *H. suis* (Florence, 1924; Żelazowska and Biliński, 1999; Hypša and Křížek, 2007).

### 16S rRNA gene sequence diversity of primary endosymbionts among populations of *Haematopinus apri* and *Haematopinus suis*

Ten insects of *H. apri* representing four collection localities in Japan (see Supplementary Table S1) were individually subjected to PCR, cloning and sequencing of bacterial 16S rRNA gene. All the sequences were completely identical to each other (accession number LC706254). DNA database searches using the sequence as query retrieved 16S rRNA gene sequences of endosymbionts of *H. suis* from United States (KX146200), *H. suis* from Czech (DQ076662), *H. apri* from Czech (DQ076665) and *H. eurysternus* from Czech (DQ076661) (Hypša and Křížek, 2007; Allen et al., 2016) as top hits. In addition, two insects of *H. suis* collected from a locality in Japan (see Supplementary Table S1) were subjected to the analysis, which yielded identical sequences (accession number LC706255) with 1 bp difference from the sequence obtained from *H. apri*. Supplementary Figure S2 A summarizes the sequence differences among the endosymbiont sequences of *H. apri* and *H. suis* from Japan, United States and Czech.

### Molecular phylogenetic analysis of primary endosymbionts of *Haematopinus apri* and *Haematopinus suis*

**Figure 2:**
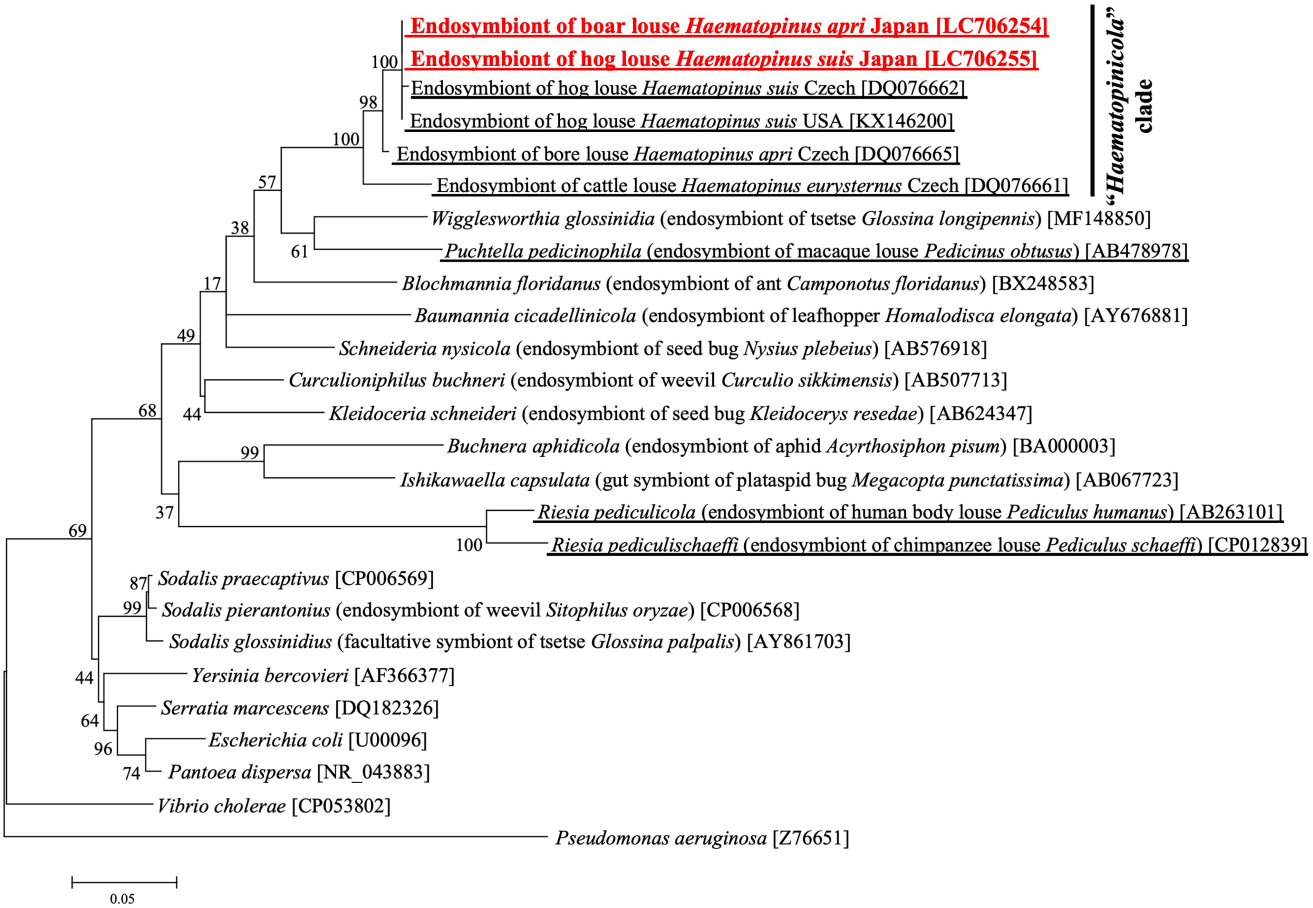
Phylogenetic placement of the primary endosymbionts of the boar louse *H. apri* and the hog louse *H. suis* based on 16S rRNA gene sequences. A maximum-likelihood phylogeny inferred from 1,013 aligned nucleotide sites is shown with bootstrap probability at each node (model: K2 + G + I). Host insect information is shown in parentheses, whereas accession number is indicated in brackets. The louse endosymbiont sequences determined in this study are highlighted in red, whereas the louse endosymbiont sequences reported in previous studies are underlined.

Figure 2 shows the phylogenetic relationship of the primary endosymbionts of *H. apri* and *H. suis* based on 16S rRNA gene sequences. The endosymbionts of *H. apri* from Japan, *H. suis* from Japan, *H. suis* from United States and *H. suis* from Czech formed a highly supported compact clade. Reflecting some nucleotide differences (see Supplementary Figure S2A), the endosymbiont of *H. apri* from Czech was placed outside the clade, and formed a highly supported clade together. Then, the endosymbiont of *H. eurysternus* was placed outside the clade of the endosymbionts of *H. suis* and *H. apri*, forming a highly supported clade together. The distinct clade of the endosymbionts of *Haematopinus* spp. was not allied to the endosymbionts of other insects including sucking lice, such as *Riesia*, *Puchtella*, *Wigglesworthia*, *Blochmannia* and *Baumannia*, in the Gammaproteobacteria.

### *In vivo* localization of primary endosymbiont of *Haematopinus apri*

**Figure 3:**
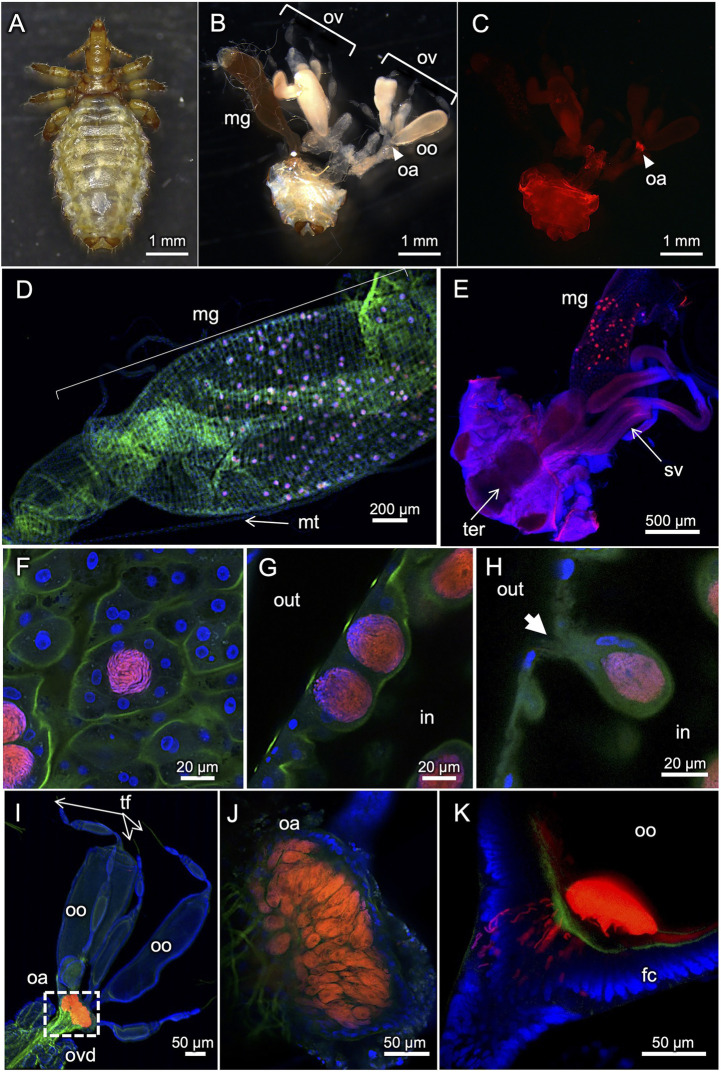
FISH visualization of *in vivo* localization of the primary endosymbiont in *H. apri*. **(A)** Dorsal view of an adult female. **(B)** Internal organs dissected from an adult female. **(C–E)** Whole-mount FISH visualization of the symbiont in dissected organs. **(C)** Localization of the symbiont in the ovarial ampulla of an adult female. The sample is the same as (**B**). **(D)** Scattered localization of the symbiont across the midgut epithelial region of an adult female. **(E)** Similar localization pattern of the symbiont in the midgut epithelium of an adult male. **(F–H)** Magnified confocal images of the symbiont in the midgut epithelium. **(F)** Optical section image parallel to the epithelial plane. **(G)** Optical section image perpendicular to the epithelial plane. **(H)** Optical section image crossing the epithelial plane, in which a bacteriocyte-like structure with an external pit is seen. The bacteriocyte-like structures are actually extracellular cavities round in shape, full of tubular bacterial cells, and surrounded by the midgut epithelial cells. **(I–K)** Localization of the symbiont in ovaries and oocytes. **(I)** Localization of the symbiont in the ovarial ampulla located at the interface of lateral oviduct and oocyte-containing ovarioles. **(J)** Optical section image of the ovarial ampulla, in which peculiar host cells are densely populated by the symbiont cells. **(K)** Optical section image of the posterior pole of an oocyte, to which the tubular symbiont cells are infecting through the follicle cell layer. Abbreviations: fc, follicle cell layer; hg, hindgut; mg, midgut; mt, Malpighian tubule; oa, ovarial ampulla; oo, oocyte; ov, ovary; ovd, oviduct; sv, seminal vesicle; ter, tergite; tf, terminal filament. In **(B,C)**, arrowheads indicate the location of the ovarial ampulla. In **(G,H)**, “out” and “in” indicate outside and inside of the midgut, respectively. In **(H)**, an arrow highlights a pit associated with the bacteriocyte-like structure.

Figure 3 shows FISH visualization of the primary endosymbiont in *H. apri*. In adult females with mature ovaries, dense symbiont signals were detected in a specialized ovarial region lying between lateral oviduct and ovarioles, so-called ovarial ampulla (Buchner, 1965) (Figures 3B,C). In both females and males, dense symbiont signals were detected throughout the midgut epithelium in a scattered manner (Figures 3D,E). Each of the patchy bacteriocyte-like structures, which contained a dense population of the symbiont cells and protruded from the epithelial wall to the midgut cavity, were actually constituted by multiple epithelial cells that encased the symbiont cells within the extracellular cavity formed at the center (Figures 3F–H). Within the ovarial ampulla, by contrast, the symbiont cells were found within the host cytoplasm endocellularly (Figures 3I,J). In mature oocytes in the ovarioles, the symbiont cells localized to the posterior pole, where infecting bacterial cells through the follicle cell layer were often observed (Figure 3K). These *in vivo* localization and infection dynamics of the primary endosymbiont were generally in agreement with previous histological descriptions on *H. eurysternus* and *H. suis* (Ries, 1931; Buchner, 1965; Żelazowska and Biliński, 1999).

### *Neisseria/Snodgrassella*-allied bacteria detected from *Haematopinus apri*

**Figure 4:**
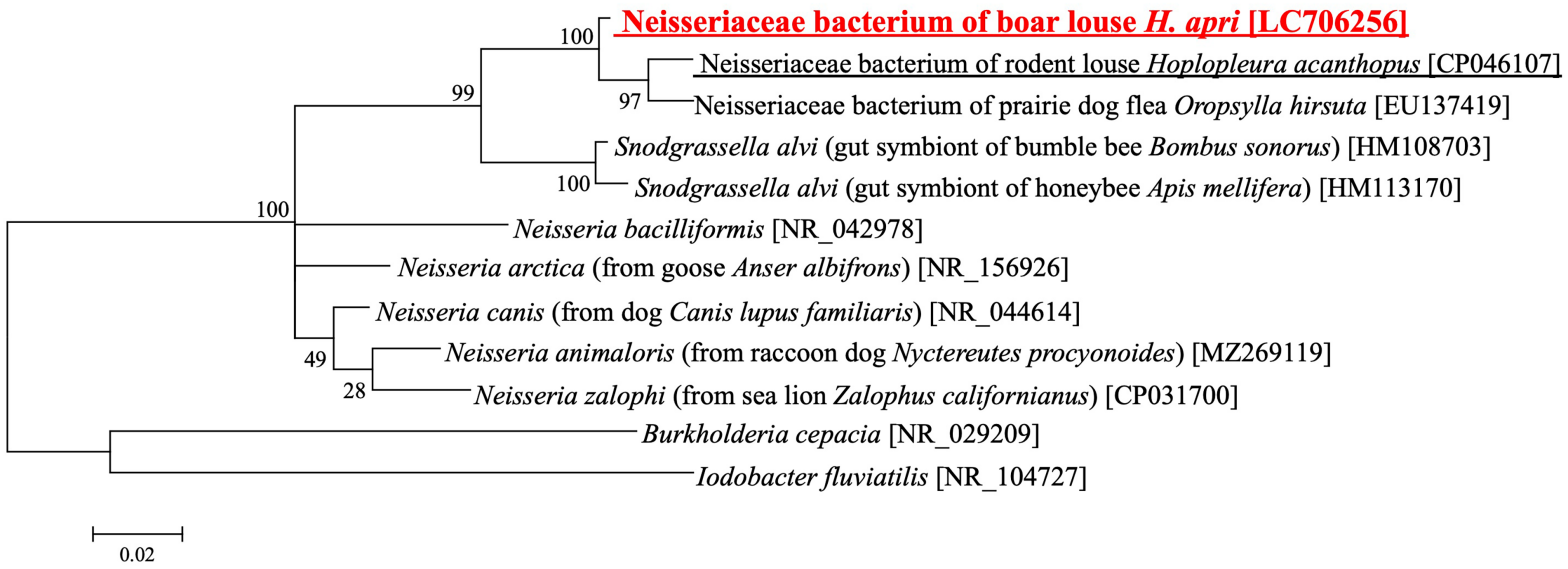
Phylogenetic placement of the *Neisseria/Snodgrassella*-allied bacterium of the boar louse *H. apri* in the Neisseriaceae based on 16S rRNA gene sequences. A maximum-likelihood phylogeny inferred from 1,063 aligned nucleotide sites is shown with bootstrap probability at each node (model: HKY + G + I). Host insect information is shown in parentheses, whereas accession number is indicated in brackets. The louse derived sequence determined in this study is highlighted in red, whereas the louse derived sequence reported in a previous study is underlined.

Recently, microbiome survey of *Polyplax* and *Hoplopleura* rodent lice identified *Neisseria/Snodgrassella*-allied bacteria as either obligatory or facultative microbial associates (Říhová et al., 2021). Since our amplicon sequencing analysis also detected *Neisseria*-allied bacterial associates from several *H. apri* samples at low frequencies (Figure 1), we cloned and sequenced 16S rRNA gene sequence of the bacteria from *H. apri*. DNA database searches using the sequence as query retrieved 16S rRNA gene sequences of Neisseriaceae bacteria from a rodent louse *Hoplopleura acanthopus* (CP046107) and a prairie dog flea *Oropsylla hirsute* (EU137419) as the top hits, and *Snodgrassella alvi* from bumble bees and honeybees (HM108703, HM113170 and others) as the next best hits. Figure 4 shows the phylogenetic relationship of *Snodgrassella, Neisseria* and allied 16S rRNA gene sequences, which indicated that the bacterium associated with *H. apri* is certainly placed in the Neisseriaceae, and the most closely related to the bacterial associates of the rodent louse *Hoplopleura* and the prairie dog flea *Oropsylla*.

### Population genetics of *Haematopinus apri* and *Haematopinus suis*

**Figure 5:**
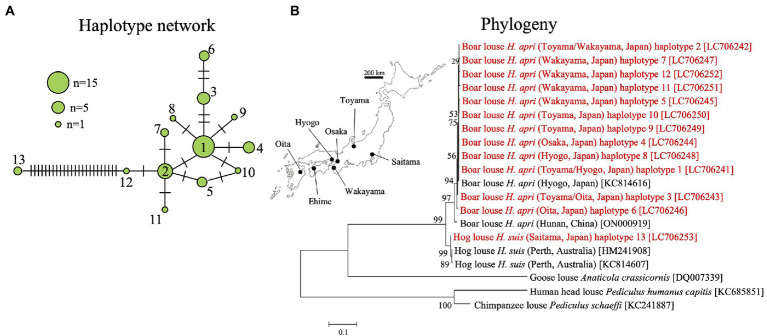
**(A)** Haplotype network of mitochondrial COI gene haplotypes representing 44 individuals from five collection localities of *H. apri* and two individuals from a collection locality of *H. suis*. **(B)** Phylogenetic relationship of the COI haplotypes of *H. apri* and *H. suis*. COI gene sequences of *H. apri* from Japan and China, and those of *H. suis* from Australia, were retrieved from the DNA databases and analyzed together. A maximum-likelihood phylogeny inferred from 516 aligned nucleotide sites is shown with bootstrap probability at each node (model: HKY + G + I). Collection locality information is shown in parentheses, whereas accession number is indicated in brackets. The collection localities are depicted on the map of Japan. The sequences determined in this study are highlighted in red.

In order to understand the population genetic structure of the host lice, mitochondrial COI gene was amplified by PCR and sequenced for 44 individuals of *H. apri* originating from five collection localities and also for two individuals of *H. suis* (see Supplementary Table S1). In total, 13 haplotypes were identified for the COI gene sequences: haplotypes 1–12 represented *H. apri* and closely related to each other, whereas haplotype 13 represented *H. suis* and was distant from the other haplotypes (Figure 5A). Supplementary Figure S2 B summarizes the sequence differences among mitochondrial COI gene sequences of *H. apri* and *H. suis* from Japan, China and Australia. In the DNA databases, a COI gene sequence of *H. apri* from Hyogo, Japan (KC814616), a COI gene sequence of *H. apri* from Hunan, China (ON000919), and two COI gene sequences of *H. suis* from Perth, Australia (HM241908, KC814607), were deposited. We performed molecular phylogenetic analysis of these COI gene sequences, and demonstrated that *H. apri* from Japanese and Chinese populations and *H. suis* from Japanese and Australian populations formed compact and well-supported sister clades, respectively (Figure 5B).

## Discussion

In the recognized diversity of the symbiotic organs and the endosymbiotic bacteria among sucking lice (Ries, 1931; Buchner, 1965; Rio et al., 2016; Husnik, 2018), previous studies on the endosymbiotic system in *Haematopinus* ungulate lice were restricted to early histological descriptions on *H. suis* and *H. eurysternus* (Florence, 1924; Ries, 1931; Żelazowska and Biliński, 1999) and a brief molecular phylogenetic characterization for *H. suis*, *H. eurysternus* and *H. apri* (Hypša and Křížek, 2007). As for the boar louse *H. apri*, the only available information on its endosymbiont has been a single 16S rRNA gene sequence (Hypša and Křížek, 2007). In this study, we investigated the endosymbiotic microbiota of *H. apri* samples derived from multiple local populations using amplicon sequencing, molecular phylogenetic and FISH histological approaches, thereby unequivocally identifying microbiological nature, *in vivo* localization and infection dynamics of the boar louse endosymbiont.

Our amplicon sequencing analysis targeting the hypervariable V3/V4 region of bacterial 16S rRNA gene identified a specific bacterial associate predominantly found from all the local populations of *H. apri* (Figure 1). Molecular phylogenetic analysis based on bacterial 16S rRNA gene sequences showed that the bacterium is closely related to and forming a distinct clade with the endosymbionts of *H. suis*, *H. apri* and *H. eurysternus* whose 16S rRNA gene sequences have been reported previously (Hypša and Křížek, 2007) (Figure 2). Detailed histological observations by FISH (Figure 3) revealed that *in vivo* localization and infection dynamics of the bacterium are concordant with the detailed early histological descriptions on the endosymbiont localization in *H. eurysternus* (Ries, 1931), and, though less comprehensive, in *H. suis* (Florence, 1924; Żelazowska and Biliński, 1999). Therefore, we conclude that this bacterium is the primary endosymbiont of *H. apri*, and suggest that this bacterial clade may represent the primary endosymbiont clade of *Haematopinus* ungulate lice.

Here, however, we point out that the endosymbiont gene sequence of Japanese *H. apri* was considerably different, by 15 nucleotide sites, from that of Czech *H. apri* (Supplementary Figure S2 A). Notably, while the endosymbiont gene sequence of Japanese *H. suis* was identical to that of American *H. suis*, the endosymbiont gene sequence of Czech *H. suis* differed by 6 nucleotide sites in comparison with that of Japanese *H. suis* (Supplementary Figure S2 A). These patterns may be explained by the following alternative hypotheses: (i) the endosymbionts of Japanese and American *H. apri* and *H. suis* are genetically differentiated from the endosymbionts of European *H. apri* and *H. suis*, respectively; or (ii) the endosymbiont gene sequences of Czech *H. apri* and *H. suis* (Hypša and Křížek, 2007) contain sequencing errors. We note that the endosymbiont sequence of Czech *H. apri* (DQ076665) contains an ambiguous (N) nucleotide site. Collection and analysis of more samples of *H. apri* and *H. suis* from Europe and other regions in the world will clarify which of these hypotheses is more appropriate.

In this study, we found that, on the basis of mitochondrial COI gene sequences, Japanese *H. apri* samples from different local populations are, though with some genetic differences, genetically coherent, whereas they are genetically distinct from Japanese *H. suis* samples (Figure 5A). Molecular phylogenetic analysis of the COI gene sequences, together with those of Chinese *H. apri* and Australian *H. suis* retrieved from the DNA databases, revealed that *H. apri* and *H. suis* are in a sister clade relationship (Figure 5B). However, considering the limited number of samples derived from the limited number of original localities examined in this study, our result should be regarded as tentative and treated with caution, with the following conditions kept in mind.

Thus far, molecular phylogenetic, comparative genomic, and archeological studies have been extensively conducted to clarify how the pig originated from the wild boar *via* domestication. These studies uncovered that (i) domestication of boars into pigs occurred at least twice independently, in East Anatolia and China, about 8,000 years ago, (ii) since then, the domestic pigs spread across the world with human movements, with occasional hybridization with local wild boars, and (iii) therefore, the genomic architecture and the evolutionary trajectory of the domestic pigs are rather mosaic and reticulated (Larson et al., 2005; Frantz et al., 2015, 2016, 2019). In this context, it is of great interest how the boar louse *H. apri*, the hog louse *H. suis*, and their endosymbionts have evolved in the process of swine domestication. The phylogenetic relationship, the species status, and the evolutionary history of *H. apri* and *H. suis* should be established with more louse samples collected from all over the world.

Besides the primary endosymbiont, amplicon sequencing analysis detected relatively minor bacterial associates of *H. apri* and *H. suis*, which were assigned as *Enterobacter*, *Citrobacter*, *Neisseria*, *Streptococcus*, *Serratia*, *Aeromonas*, *Turicibacter*, etc. (Figure 1). On account of the low incidence in the amplicon sequencing data, these bacteria are plausibly facultative and/or casual associates for the host insects. Among them, the *Neisseria-*allied bacterium associated with *H. apri* is of particular interest, because it was recently reported that *Neisseria/Snodgrassella*-allied bacteria are associated with rodent lice of the genera *Hoplopleura* and *Polyplax* as their potential endosymbionts (Říhová et al., 2021). Molecular phylogenetic analysis verified that the *Neisseria*-like bacterium of *H. apri* is closely related to Neisseriaceae bacteria from the rodent louse *H. acanthopus* and the prairie dog flea *Oropsylla hirsute*, and also to the gut symbionts of bees *Snodgrassella alvi* (Figure 4). It is also notable that *Neisseria* species tend to be associated with mammals and birds, such as *N. canis* with dog, *N. animaloris* with racoon dog, *N. zalophi* with sea lion, *N. arctica* with goose, etc. (see Figure 4), as inhabitants of the mucous membranes of animals (Tønjum, 2015). These observations suggest the possibility that, although speculative, the blood-sucking lice may acquire the *Neisseria*-allied bacteria from their host animals either casually or as commensal associates, and some of them have established an obligatory association as observed in *H. acanthopus* (Říhová et al., 2021).

On the basis of the distinct microbiological, phylogenetic and histological features described in this and previous studies, we propose the designation ‘*Candidatus* Haematopinicola symbiotica’ for the hitherto unnamed endosymbiotic bacterial clade associated with ungulate lice of the genus *Haematopinus*. The generic name highlights the endosymbiotic association with *Haematopinus* spp., and the specific name indicates the endosymbiotic nature of the bacterial clade. Thus far, the cattle louse *H. eurysternus* (Ries, 1931; Hypša and Křížek, 2007), the hog louse *H. suis* (Sikora, 1919; Buchner, 1920; Florence, 1924; Ries, 1931; Żelazowska and Biliński, 1999; Hypša and Křížek, 2007; this study) and the boar louse *H. apri* (Hypša and Křížek, 2007; this study) have been shown to host ‘*Ca.* H. symbiotica’. Histologically, the horse louse *H. asini* seems likely to harbor ‘*Ca.* H. symbiotica’ (Buchner, 1965). Whether the other *Haematopinus* species are also associated with ‘*Ca.* H. symbiotica’ requires verification in future studies.

In conclusion, we characterized the primary endosymbiont of the boar louse *H. apri* in detail, uncovered its population genetic and phylogenetic aspects in relation to host’s local populations, and proposed the designation ‘*Ca.* H. symbiotica’ for the primary endosymbiont clade of the louse genus *Haematopinus*. The biological role of ‘*Ca.* H. symbiotica’ is expected as provisioning of B vitamins for the blood-sucking host lice, which should be verified by sequencing and analysis of the endosymbiont genomes. Extensive worldwide collection of *H. apri* and *H. suis* samples and genetic analysis of them and their endosymbionts would uncover how the boar louse, the hog louse, and their endosymbionts have evolved in the process of swine domestication in the human history.

## Data availability statement

The datasets presented in this study can be found in online repositories. The names of the repository/repositories and accession number(s) can be found in the article/Supplementary material.

## Author contributions

YN performed molecular phylogenetic and amplicon sequencing analyses with support by MaM and MiM. KO conducted histological analyses with support by RK. TF conceived the study and arranged collection of insect samples. YN and TF wrote the paper. All authors contributed to the article and approved the submitted version.

## Funding

This study was supported by the Japan Science and Technology Agency (JST) ERATO Grant Numbers JPMJER1803 and JPMJER1902 to RK and TF, and by the Japan Society for the Promotion of Science (JSPS) KAKENHI Grant Number JP17H06388 to TF. KO was supported by the JSPS Research Fellowships for Young Scientists Number 21 J01321.

## Conflict of interest

The authors declare that the research was conducted in the absence of any commercial or financial relationships that could be construed as a potential conflict of interest.

## Publisher’s note

All claims expressed in this article are solely those of the authors and do not necessarily represent those of their affiliated organizations, or those of the publisher, the editors and the reviewers. Any product that may be evaluated in this article, or claim that may be made by its manufacturer, is not guaranteed or endorsed by the publisher.

## Supplementary material

SUPPLEMENTARY TABLE S1 Samples of *H. apri* and *H. suis* used in this study with sequence accession numbers.

SUPPLEMENTARY FIGURE S1 FISH detection and RNase digestion control of the primary endosymbiont in the midgut epithelium of *H. apri*.

SUPPLEMENTARY FIGURE S2 (**A**) Nucleotide differences among 16S rRNA gene sequences of the primary endosymbionts of *Haematopinus apri* and *Haematopinus suis* from Japan, United States and Czech. Number of nucleotide differences out of 1100 aligned nucleotide sites is indicated for each of the sequence pairs. Note that the sequence of *H. apri* symbiont Czech [DQ076665] contains an ambiguous (N) site. (**B**) Nucleotide substitutions among mitochondrial COI gene sequences of *H. apri* and *H. suis* from Japan, Australia and China. Number of nucleotide differences out of aligned nucleotide sites is indicated for each of the sequence pairs. The sequences determined in this study are highlighted in red.
