# Supplementary figures and images for: Endosymbiotic bacteria of the boar louse Haematopinus apri (Insecta: Phthiraptera: Anoplura)
Source: Front Microbiol. 2022 Aug 8;13:962252. doi: 10.3389/fmicb.2022.962252 (PMC9393614; doi:10.3389/fmicb.2022.962252)

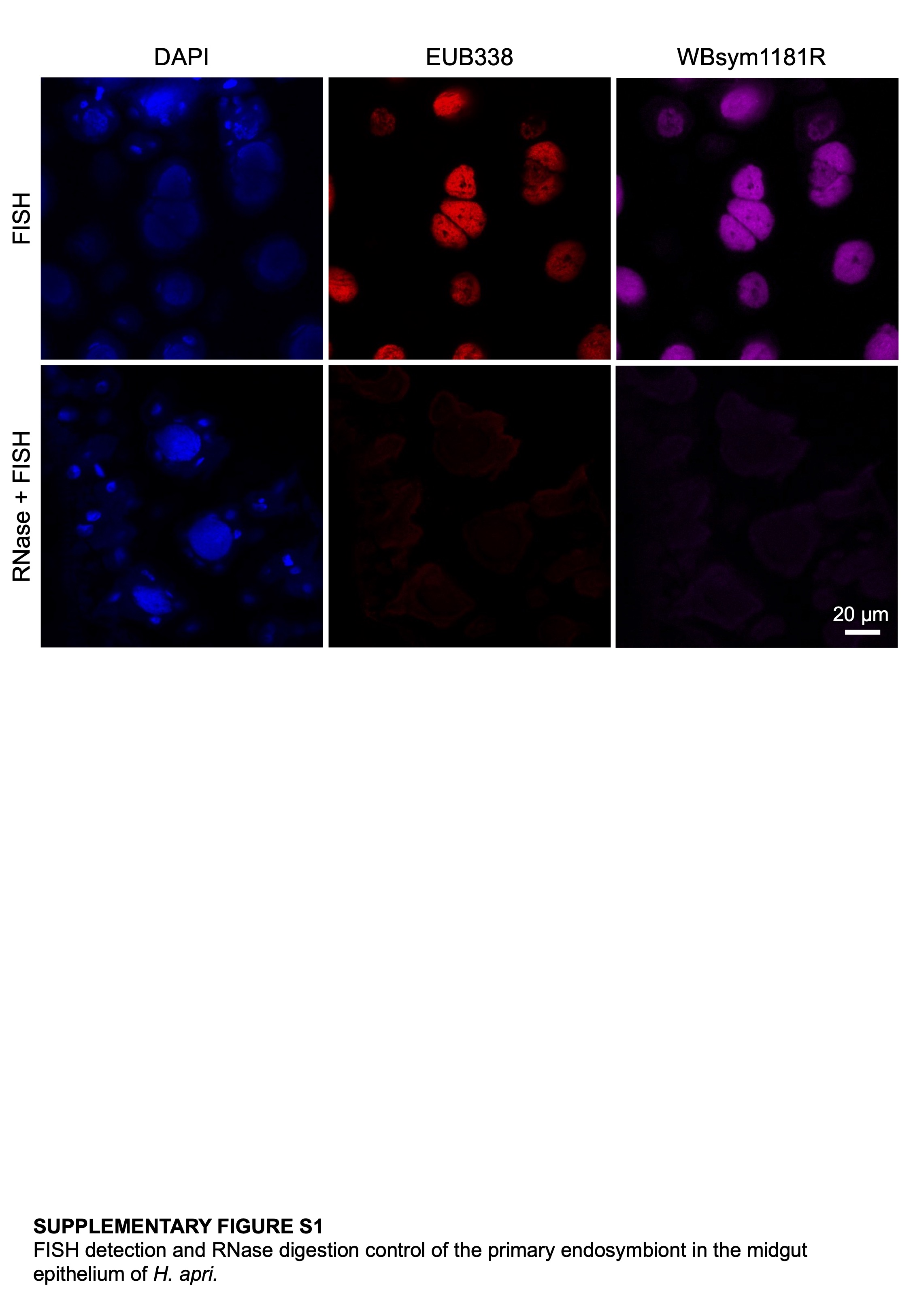

Supplement: SUPPLEMENTARY FIGURE S1 — FISH detection and RNase digestion control of the primary endosymbiont in the midgut epithelium of H. apri. [file Image_1.jpeg]

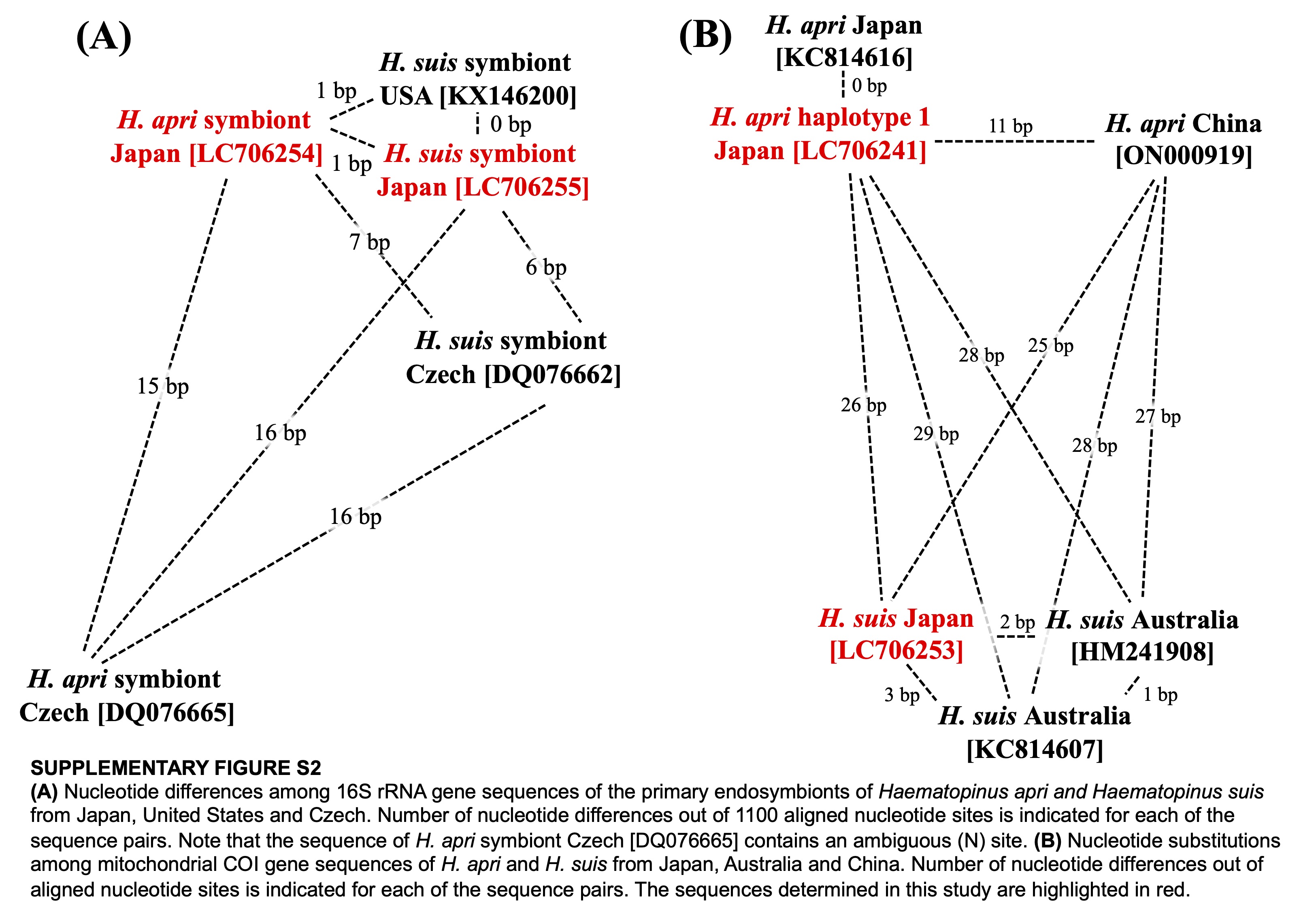

Supplement: SUPPLEMENTARY FIGURE S2 — (A) Nucleotide differences among 16S rRNA gene sequences of the primary endosymbionts of Haematopinus apri and Haematopinus suis from Japan, United States and Czech. Number of nucleotide differences out of 1100 aligned nucleotide sites is indicated for each of the sequence pairs. Note that the sequence of H. apri symbiont Czech [DQ076665] contains an ambiguous (N) site. (B) Nucleotide substitutions among mitochondrial COI gene sequences of H. apri and H. suis from Japan, Australia and China. Number of nucleotide differences out of aligned nucleotide sites is indicated for each of the sequence pairs. The sequences determined in this study are highlighted in red. [file Image_2.jpeg]
